# Artificial Intelligence Bias in Health Care: Web-Based Survey

**DOI:** 10.2196/41089

**Published:** 2023-06-22

**Authors:** Carina Nina Vorisek, Caroline Stellmach, Paula Josephine Mayer, Sophie Anne Ines Klopfenstein, Dominik Martin Bures, Anke Diehl, Maike Henningsen, Kerstin Ritter, Sylvia Thun

**Affiliations:** 1 Core Facility Digital Medicine and Interoperability Berlin Institute of Health at Charité – Universitätsmedizin Berlin Berlin Germany; 2 Institute for Medical Informatics Charité – Universitätsmedizin Berlin Berlin Germany; 3 Stabsstelle Digitale Transformation Universitätsmedizin Essen Essen Germany; 4 Faculty of Health University of Witten/Herdecke Witten Germany; 5 Department of Psychiatry and Psychotherapy Charité – Universitätsmedizin Berlin Berlin Germany

**Keywords:** bias, artificial intelligence, machine learning, deep learning, FAIR data, digital health, health care, online, survey, AI, application, diagnosis, treatment, prevention, disease, age, gender, development, clinical

## Abstract

**Background:**

Resources are increasingly spent on artificial intelligence (AI) solutions for medical applications aiming to improve diagnosis, treatment, and prevention of diseases. While the need for transparency and reduction of bias in data and algorithm development has been addressed in past studies, little is known about the knowledge and perception of bias among AI developers.

**Objective:**

This study’s objective was to survey AI specialists in health care to investigate developers’ perceptions of bias in AI algorithms for health care applications and their awareness and use of preventative measures.

**Methods:**

A web-based survey was provided in both German and English language, comprising a maximum of 41 questions using branching logic within the REDCap web application. Only the results of participants with experience in the field of medical AI applications and complete questionnaires were included for analysis. Demographic data, technical expertise, and perceptions of fairness, as well as knowledge of biases in AI, were analyzed, and variations among gender, age, and work environment were assessed.

**Results:**

A total of 151 AI specialists completed the web-based survey. The median age was 30 (IQR 26-39) years, and 67% (101/151) of respondents were male. One-third rated their AI development projects as fair (47/151, 31%) or moderately fair (51/151, 34%), 12% (18/151) reported their AI to be barely fair, and 1% (2/151) not fair at all. One participant identifying as diverse rated AI developments as barely fair, and among the 2 undefined gender participants, AI developments were rated as barely fair or moderately fair, respectively. Reasons for biases selected by respondents were lack of fair data (90/132, 68%), guidelines or recommendations (65/132, 49%), or knowledge (60/132, 45%). Half of the respondents worked with image data (83/151, 55%) from 1 center only (76/151, 50%), and 35% (53/151) worked with national data exclusively.

**Conclusions:**

This study shows that the perception of biases in AI overall is moderately fair. Gender minorities did not once rate their AI development as fair or very fair. Therefore, further studies need to focus on minorities and women and their perceptions of AI. The results highlight the need to strengthen knowledge about bias in AI and provide guidelines on preventing biases in AI health care applications.

## Introduction

Due to the growing amount of health data combined with the desire to gain ground in precision medicine, artificial intelligence (AI) is advancing at a rapid pace across the health care sector [1]. AI applications in medicine enable the analysis of a wide variety of health data types, ranging from web-based applications using machine learning (ML) to physical applications such as intelligent prostheses [2] and sophisticated robots [3]. ML is a subset of AI using large data inputs and outputs with the goal of recognizing patterns leading to autonomous recommendations or decisions. It can be categorized as follows: unsupervised (ability to find patterns), supervised (based on previously provided labels), and reinforcement learning (sequences of rewards and punishments) [4]. Deep learning (DL), another subset of AI, is a class of ML models using artificial neural networks to learn complex relationships between features and labels operating directly on raw or minimally processed data [5]. In a health care setting, AI models require substantial data with highly comprehensive and, in some cases, longitudinal patient information. However, health data sets generally lack a common structure, format, and standardization [6]. In addition, data are not generally integrated across all health care providers but exist relatively isolated in electronic health care records and are therefore prone to being biased [7].

To derive the greatest use of medical AI applications, algorithms must be fair, meaning key population characteristics that impact algorithm outcomes and target variables are considered in the algorithm. Bias in AI, also called algorithmic bias, can be described as an ML model yielding a systematically wrong outcome [8] because of the differential consideration of certain informational aspects, such as gender, age, or ethnic group, contained in a data set [9]. There are already documented examples of biases in AI applications such as facial recognition, in which algorithms perform poorly with faces of females or Black individuals [10], or natural language processing, in which human-like gender biases occur [8,9,11]. A major problem for algorithms used on health data is the distributional shift, meaning a mismatch between training and test data leading to erroneous predictions. When there is bias in the test data and therefore not correct representations of the general population, known inherent variations in the population are not correctly reflected in the resulting output [12].

In particular, the influence of sociocultural gender and biological sex on health conditions is often ignored in algorithm design and study data. One example is the algorithm predicting acute kidney injury, which was trained on a data set containing only 6% females and hence had lower performance among that demographic subgroup [13]. However, sex- and gender-specific differences have been described in several pathological and physiological processes [14-18]. Even recently, it was shown that only 18% of clinical trials on COVID-19 registered on ClinicalTrials.gov and published in scientific journals reported sex-disaggregated results or subgroup analysis [19]. Despite potential biases, defined methods for bias detection and prevention are not officially mandated within the development of AI applications, despite their existence. Explainable artificial intelligence (XAI) refers to algorithms that meet interpretability and completeness requirements [20]. XAI aims to establish transparency by explaining what decisions led to the creation of the algorithm in addition to its inputs and outcomes, which provide the basis for trusting the algorithm [21]. Methods through which XAI can be established include layer-wise relevance propagation [22] and rationalization [23]. Furthermore, Friedrich et al [24] discussed the role and benefits of statistics, which they see as a natural partner in AI developments, for example, in calculating the sample size but also for bias control. As the existence of biases in AI is a known fact, little is known about the perception and knowledge of AI developers on biases and their prevention. This study set out to answer the following research questions from the AI developers’ perspective: (1) How fair are current AI developments? (2) What is preventing fair AI algorithms? and (3) What data are used to train AI algorithms?

## Methods

### Survey Design

Survey development was initiated after a literature review to establish an understanding of the current state of knowledge on bias in AI algorithms and after consultation with several experts in the fields of bias and AI development who signed on as coauthors. The team of the Berlin Institute of Health (BIH), comprised of medical doctors with expertise in interoperability and digital medicine, drafted all survey questions in collaboration with the partners.

The final survey (version 5) was the result of an iterative process of developing questions that comprised the first 4 survey versions, consulting experts for review and validation in each instance, and making adjustments in the wording and the question design. The first questionnaire consisted of 29 questions without adaptive questioning. The final web-based questionnaire contained 4 routing and completeness variables, 22 base questions, and 15 questions that would appear based on specific answer choices. The final set of questions included 5 new ones compared to the first version, added to gather demographic details (age and workplace) and qualitative insights into the familiarity of respondents with bias prevention measures. All survey questions except for 3 (1 dichotomous question and 2 Likert-scale questions) were designed to collect qualitative data. Overall, the survey that went live had a maximum of 41 questions, using adaptive questioning to reduce the complexity and volume of questions. Single- and multiple-choice questions were included, as were free-text fields for further explanatory comments. Categorical questions included a “not specified” option to select for nonresponse. The questionnaire was developed both in German and English and can be found in Multimedia Appendix 1.

Study data were collected and managed using REDCap (Vanderbilt University) tools hosted at Charité - Universitätsmedizin Berlin [25,26]. On the survey landing page, participants were informed about the survey goals, its length, the target group, and the investigator organizations in both English and German. After choosing the survey language, participants were redirected to the first survey section. Participation in this anonymous study was voluntary, and no incentives were provided. The first survey page included demographical questions regarding country, institution, gender, age, and work environment. The participant could only proceed to and answer the next section of the questionnaire if they answered that they had experience in AI development; otherwise, the survey ended at this point. The second survey page focused on the type of AI development and medical specialty the participants were involved in. Respondents were able to review and change answers by clicking on the “Previous Page” button shown on the survey screen. The comprehensibility, usability, and technical functionality of the survey were tested by several employees of the inquiring institutions prior to its launch. We used REDCap’s internal functionality to check the completeness of each questionnaire page after a participant had submitted it.

### Code Availability

All R scripts that were written and used for analysis in this study can be accessed via a dedicated GitHub repository, which is referenced in the data availability statement.

### Recruitment

The target group of the survey was participants with experience in developing AI applications in health care. The survey was distributed on the web as an open survey inviting voluntary participation. Announcements were made through the newsletters of universities, other institutions, associations, and organizations in digital health, through mailing lists, as well as by contacting professionals directly via email. In addition, social media accounts (LinkedIn and Twitter) and web magazines were used to recruit participants. An overview of the wording used to announce the survey and invite participation is shown in Multimedia Appendix 2. We provided a dedicated URL leading to the survey via a link. The survey was kept open from August 20, 2021, to November 20, 2021.

### Data Exclusion

**Figure 1:**
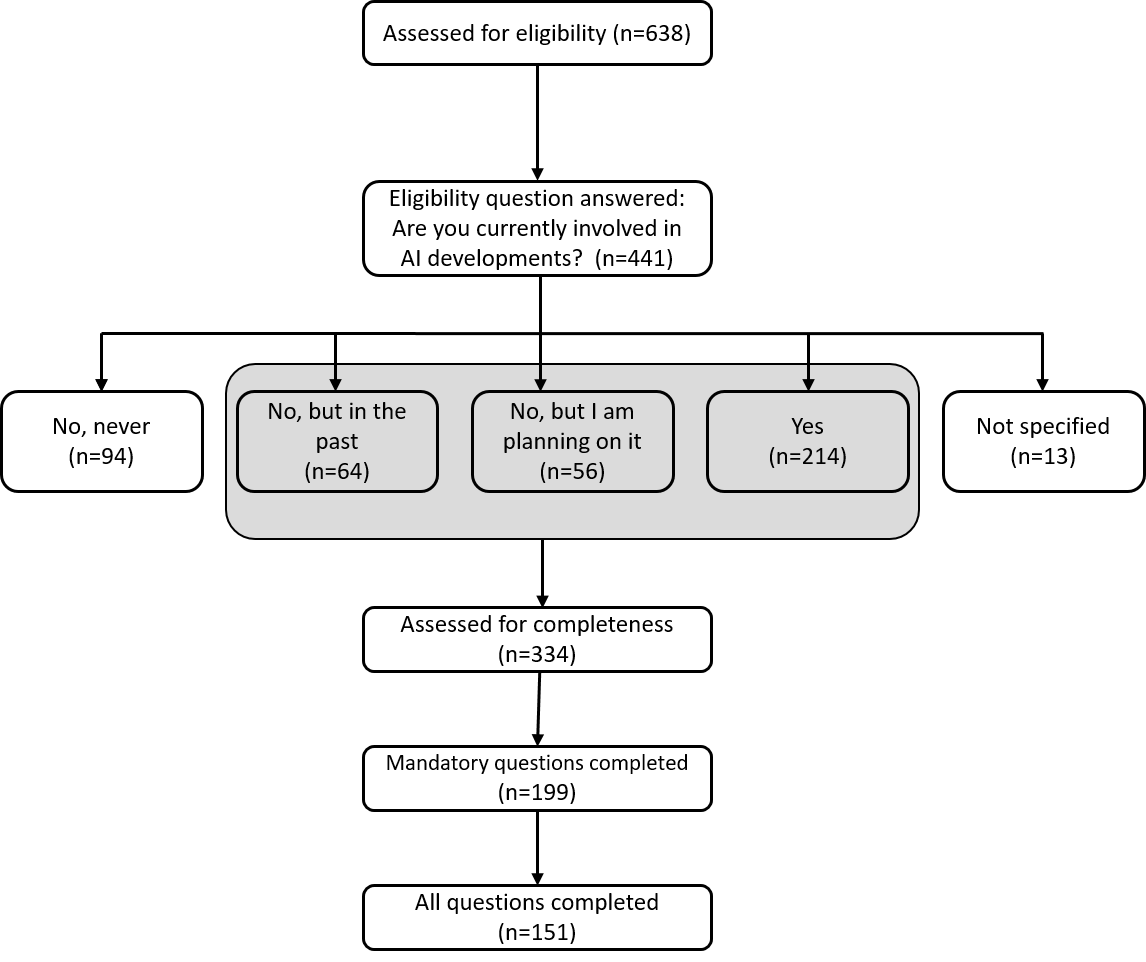
Flow diagram outlining the number of eligible and responding participants to the survey, as well as the number of participants included in the analysis. AI: artificial intelligence.

The primary criteria for inclusion in this study were the responses to the question, “Are you currently involved in AI developments?” Only entirely completed questionnaires from participants with experience in AI development were included. The process of data exclusion is demonstrated in Figure 1.

### Data Analysis

Tables and Likert graphs were used to present the data synthesis results. For categorical demographic variables, simple and relative frequency and proportions were used. Continuous variables are presented in median and IQR unless otherwise stated. Categories for age groups were created based on the distribution of data. For reproducibility purposes, the survey data were downloaded from REDCap directly into an R project, and analysis of the survey data was performed using RStudio (version 1.4.1106; R Studio, PBC).

### Ethical Considerations

All participants provided consent and were provided with the survey duration and purpose of the study prior to the survey. No personal information was collected or stored. There were no incentives offered to complete the survey. According to the “Ärztliche Berufsordnung, page 8, §15 Forschung (1),” ethical approval for this study was not requested.

## Results

### Demographics

A total of 638 participants answered the survey and were assessed for eligibility. Of the 441 participants who answered the eligibility question, 107 were excluded due to a lack of experience in AI development. After further exclusion of 183 incomplete questionnaires, a total of 151 participants involved in AI development completed the survey with a median age of 30 (IQR 26-39) years. The majority of respondents worked in Germany (139/151, 92%), while 2% (3/151) worked in the United States and less than 1% (1/151) in Austria, the Czech Republic, Finland, France, Hungary, the Netherlands, Spain, Switzerland, and Scotland, respectively. Participants received the survey via email distribution (72/151, 48%), personal contact (25/151, 17%), LinkedIn (25/151, 17%), Twitter (11/151, 7%), other options (16/151, 11%), or did not specify the channel of survey reception (2/151, 1%). Details on demographics are found in Table 1 and Table S2 in Multimedia Appendix 2.

**Table 1:** Demographics of survey participants (N=151).

Characteristics		Participants, n (%)
**Gender**		
	Male	101 (67)
	Female	45 (30)
	Undefined	2 (1)
	Not specified	2 (1)
	Diverse	1 (1)
**Age (years)**		
	≤30	69 (46)
	30-40	49 (33)
	41-50	24 (16)
	≥50	9 (6)
**Work environment**		
	Science	104 (69)
	Industry	22 (15)
	Clinical work	12 (8)
	Other	11 (7)
	Not specified	2 (1)
**Most common medical specialties**		
	Digital medicine	38 (25)
	Radiology	32 (21)
	Not specified	29 (19)
	Other	22 (15)
	Internal medicine	21 (14)
	Surgery	14 (9)
	Family medicine	14 (9)
	Public health	11 (7)
	Neurology	10 (7)
**Stage of AI^a^ project**		
	Training and optimization	105 (70)
	Data acquisition or preprocessing	84 (56)
	Identification of AI algorithms	83 (55)
	Project planning	82 (54)
	Practical testing of AI algorithms	79 (52)
	Data annotation	61 (40)
	Not specified	7 (5)
	Other	6 (4)

^a^AI: artificial intelligence.

### AI Experience of Participants

The majority (105/151, 70%) of participants used ML within their AI project, followed by DL (86/151, 57%) and other types of AI (41/151, 27%). Among participants working with ML, 53% (80/151) used supervised ML, 28% (42/151) semisupervised ML, 27% (41/151) unsupervised ML, and 14% (21/151) reinforcement learning. Five percent (7/151) of respondents used other ML techniques and 4% (6/151) did not specify their answer. Participants working with DL used convolutional networks (71/151, 47%), recurrent neural networks (40/151, 26%), autoencoders (30/151, 20%), and other types of DL (26/151, 17%). Participants used natural language processing (39/151, 26%), clinical decision support (53/151, 35%), image processing (64/151, 42%), computer vision (50/151, 33%), and robotics (16/151, 11%) within their AI developments.

### Current Knowledge of Biases in AI

Most respondents (113/151, 75%) had heard of biases before and knew specific use cases, while 20% (30/151) of respondents could not think of concrete examples. Five percent (8/151) had never heard of biases in AI before. When asking respondents where they think biases in AI could possibly occur, the majority voted for societal factors (126/151, 83%), followed by the methodology of algorithms (99/151, 66%), data validation, or data security (119/151, 79%). No respondent answered that none of these options could provide biases, and 12% (18/151) felt that there were other parameters that could lead to biases in AI. Table 2 presents the knowledge distribution in terms of preventive measures to avoid biases in AI.

**Table 2:** Do you know any of the following preventive measures to avoid bias in AI applications?

Knowledge of preventive measures	Participants, n (%)
XAI^a^	85 (56)
Collecting sociodemographic data	53 (35)
Statistical analysis	95 (63)
Software evaluating fairness in AI^b^	44 (29)
I do not know any of them	25 (17)
Other	3 (2)

^a^XAI: explainable artificial intelligence.

^b^AI: artificial intelligence.

### Data Used in AI Projects

Half of the respondents worked with image (83/151, 55%) and text data (77/151, 51%). Audio data were used by 12% (18/151) of respondents, and 16% (24/151) of respondents did not specify the type of data used in their AI projects. Regarding the origin of the data, half of the respondents used data from 1 center (76/151, 50%). Multicenter databases (50/151, 33%), registries (25/151, 17%), and wearables (18/151, 12%) were also used for AI algorithms. Other or unspecified data sources were used by 21% (32/151) and 12% (18/151) of respondents, respectively. Thirty-five percent of respondents (53/151) used national data only, 33% (50/151) used national and international data, and 13% (19/151) worked with international data only.

### Prevention of Biases in AI

**Figure 2:**
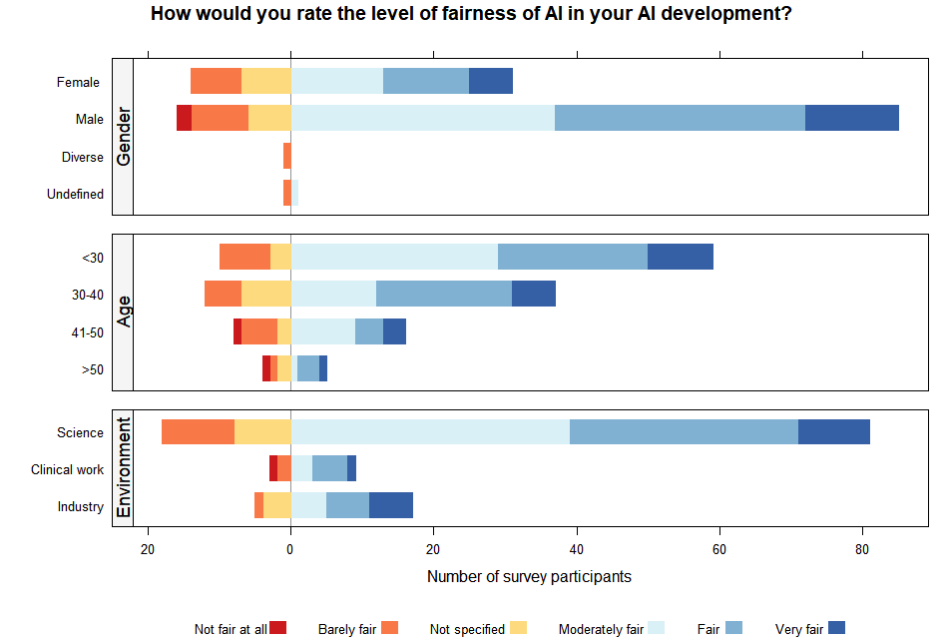
Likert graphs displaying the perception of fair AI among AI developers. The horizontal axis corresponds to the absolute number of survey participants, while the vertical axis details the absolute response counts. The level of fairness was shaded in colors, as detailed in the figure legend. AI: artificial intelligence.

When asked whether standardized data by using international semantic and syntactic standards such as HL7 FHIR or SNOMED CT could reduce bias in AI, only 25% (37/151) answered “yes,” 44% (66/151) answered “no,” and 32% (48/151) did not specify their answer (Figure 2). Regarding including sociodemographic information in training data for AI algorithms, most of the respondents (100/151, 66%) would collect data on age to prevent biases, followed by biological gender (95/151, 63%) and origin (95/151, 63%). Social gender was chosen by 54% (81/151) of participants, and 6% (9/151) would collect none of the suggested data points. When asked what the participants would use the sociodemographic data for, 62% (93/151) said they would use it for analysis of data, while 38% (57/151) would use the data for AI modeling and 40% (61/151) for data acquisition.

### Current Perception of Biases in AI

When the entire cohort was asked how they would rate the level of fairness of AI in their own AI development, one-third rated their development as fair (47/151, 31%) or moderately fair (51/151, 34%). AI developments were rated as very fair by 13% (19/151) of respondents. Twelve percent (18/151) gave a rating for barely fair and 1% (2/151) for not fair at all. Among the 132 (87%) respondents who did not rate their project as very fair, possible reasons preventing fair AI were explored: the majority of respondents selected lack of fair data (90/132, 68%), followed by lack of guidelines or recommendations (65/132, 49%), as well as lack of knowledge (60/132, 45%). Lack of support from superiors, institutions, or other options was answered by 9% (12/132) and 5% (7/132), respectively.

### Level of Fairness Perception by Gender, Age Group, and Work Environment

Figure 2 shows the perception of fairness by gender, age group, and work environment. We defined fairness as whether the decision-making process of the algorithm takes sensitive factors like gender, race, and age into account, meaning whether specific groups of people are treated or considered differently from others [27]. There was a significant difference among participants with regard to the work environment (*P*=.02): 5% (1/22) of respondents working in the industry compared to 10% (10/104) working in science, and 25% (3/12) of respondents working in a clinical setting rated their AI developments as not fair at all or barely fair. There was no significant difference in the level of fairness expressed by respondents in terms of their gender (*P*=.17) or age group (*P*=.09). The 3 participants identifying as diverse or undefined rated their AI development as barely fair (1/1) or barely fair (1/2) and moderately fair (1/2), respectively, while male participants had the highest percentage of a very fair perception (13/101, 13%).

## Discussion

### Principal Findings

Biased algorithms in health care are not news; however, established guidelines, laws, or literature on the practical use of fair AI algorithms in health care are scarce [28]. Therefore, we investigated whether AI developers perceived AI algorithms as fair, as well as their knowledge of preventive measures. We found that one-third of participants rated their AI project as either fair or moderately fair, while 13% (20/151) rated their AI project as barely fair or not fair at all. The main reasons for biases were a lack of fair data, guidelines, and recommendations, as well as knowledge of biases in AI. There was no difference in bias perception among different genders; however, participants did not represent a diverse overall population. The majority of respondents were male, younger than 35 years, and employed in a scientific work environment. Half of the respondents worked with image data from 1 center only, and one-third worked with national data only.

Most AI projects were in the current stage of training and optimization, while only 52% (79/151) performed practical testing of AI algorithms. This could also introduce bias into our results, as possible AI biases might occur during testing when models perform poorly in data sets that differ from the training data [13,29]. When asked about preventive measures regarding biases in AI, more than half of the participants were aware of methods such as XAI and statistical analysis. Less than half of the participants were aware of collecting sociodemographic data or software evaluating fairness in AI, highlighting the need for education on these methods. Interestingly, 17% (25/151) of participants did not know any of these preventive measures, and 5% of AI developers had never heard of biases in AI before calling for general training to prevent biases in AI.

The data types most commonly used for AI projects were image data, followed by text data. This coincides with the fact that the second most common medical specialty after digital medicine was the field of radiology, in which survey participants worked. In terms of data availability, most data originated from 1 center only in a national setting. One-third used international data in addition to national data, and a small proportion worked with international data only. This might be concerning as the increase in training set size can reduce discrimination within AI [30].

Only one-quarter of participants felt that using international standards could reduce biases, despite interoperability being one of the main parts of the FAIR data principles, meaning findable, accessible, interoperable, and reusable [28,31]. Interoperability describes the ability of systems to exchange data and use the data after these have been received [32]. While there are several levels of data interoperability (semantic, syntactic, organizational, etc) [33], the prevention of bias in AI can be assisted through the use of standard terminologies and ontologies. International standard codes provide an unambiguous meaning to health care concepts such as diagnosis, drug prescriptions, and demographic parameters present in data and can facilitate semantic interoperability [34,35].

Overall, developers participating in this study supported the inclusion of sociodemographic parameters. The 3 characteristics of age, biological sex, and ethnicity were most supported by respondents. The significance of including such data for the purpose of data acquisition and analysis as well as AI modeling should not be underestimated. All categories influence many physiological and biochemical processes and can drive pathogenic and homeostatic phenotypes [36], important variables that AI models aim to predict or model. Social gender would have been collected by more than half of the respondents. However, most respondents would use this sociodemographic data more for analysis than AI modeling.

The fact that lack of fair data was named as the main reason for reported shortcomings in terms of fairness highlights the potential and importance of establishing fair and accessible training data for the development of algorithms. In addition, the lack of usable guidelines or recommendations for establishing fairness in AI was mentioned as the second obstacle to fair AI. This highlights a clear need for guidance on how fair AI can technically be implemented and achieved in AI health care applications. Arguably, such guidance could best be provided by accredited international institutions with strong competencies in the fields of AI and health care, as well as an excellent understanding of the FAIR data principles [31]. Recent publications have presented approaches defining ethical and regulatory boundaries for AI applications in health care, including a governance model [37], a bias evaluation checklist for predictive models used in hospital settings [38], and an overview of ethical principles to be considered in the regulation of bias in health care ML [39]. Furthermore, in July 2018, the World Health Organization established the Focus Group on “Artificial Intelligence for Health” together with the International Telecommunication Union. The Focus Group on “Artificial Intelligence for Health” aims to identify concerns in health care AI regarding data, processes, and algorithms and to develop guidelines while creating a web-based platform and benchmarking tools for health AI [40].

When asking participants whether they felt their AI development was perceived as fair, 13% (19/151) of respondents rated their work as very fair, while approximately 65% (98/151) of participants considered their projects fair or moderately fair. However, the majority of respondents in this survey cohort were male. As the participants were not as diverse when mostly represented by male AI developers, these results could be skewed, and a future aim would be to specifically target opinions by social minorities regarding health data. This is supported by the fact that the 3 participants in our survey who identified as diverse or undefined rated their AI development as barely fair or moderately fair only. Among AI specialists in health care, especially in Germany, one of the leading AI countries, there is an underrepresentation of women, as only 24% of AI specialists are female [41]. The fact that AI specialists are not prone to diversity does not only relate to gender but also race and other underrepresented groups and should be targeted, as including diverse stakeholders was previously reported to be a recommendation for incorporating fairness into AI [29]. A future aim would be to specifically seek out the opinions of social minorities and women regarding fairness in health data AI through a targeted questionnaire that could be distributed using social media and professional networks.

When investigating the perception of fairness among work environments, we found that the majority of survey participants in a scientific work environment rated their AI development only as moderately fair, while the majority from the industry evaluated their application as fair or even very fair. Developers in clinical work were the only ones that rated their algorithms as not fair at all, and they also constituted the highest subgroup that rated algorithms as barely fair. The survey data do not point to a clear explanation for this observation. When it comes to data in health care, the fact that especially hospital information systems were not created to allow for modern analytics and the lack of full integration with other relevant external and internal data systems present a significant challenge [8]. In addition, data generated in experimental, preclinical, or clinical research settings that may serve as the basis for developing algorithms often contain inherent sex biases due to the overrepresentation of male study subjects over females. When working with a limited budget and resources such as time and workforce, ensuring fairness in AI might be neglected in favor of using resources for other purposes due to the lack of importance attributed to it. A more in-depth follow-up study would aid in investigating this further.

### Limitations and Outlook

While the power of this study to draw conclusions might be limited due to the relatively small sample size [42], the survey results provide a first set of insights into the use of fair data in AI development for health care use cases. Future studies will have to focus on addressing a larger AI developer audience in order to reduce response bias [43]. As the majority of participants worked in Germany and were mostly male, the survey may not be generalizable. Furthermore, another limitation is the web-based nature of this survey, which lacks a formal sampling frame. Since the majority of survey questions were designed to collect qualitative data, the determination of reliability and validity of questions using measures such as Cronbach α [44], which require the presence of at least 3 quantitative questions, cannot be performed. Issuing a second, more targeted, and structured survey in a year’s time would allow for a follow-up probe into how the field is evolving in industry versus a scientific and clinical setting. The responses from this study have shed new light on developers’ awareness of bias in AI and showed that there is a need for education on preventive measures, especially with regard to fair data and the FAIR principles, as well as including sociodemographic factors for training AI algorithms. Guidelines and recommendations are warranted to guarantee fair algorithms that are generalizable to the target population.

## Appendix

Multimedia Appendix 1 Questionnaire.

Multimedia Appendix 2 Supplementary tables.

## Abbreviations

AI: artificial intelligence
BIH: Berlin Institute of Health
DL: deep learning
ML: machine learning
XAI: explainable artificial intelligence

## Data Availability

The survey protocol, data, and analysis scripts can be accessed via a dedicated GitHub repository [45].

## References

[1] Jiang F, Jiang Y, Zhi H, Dong Y, Li H, Ma S, Wang Y, Dong Q, Shen H, Wang Y (2017). Artificial intelligence in healthcare: past, present and future. Stroke Vasc Neurol.

[2] Fajardo J, Maldonado G, Cardona D, Ferman V, Rohmer E (2021). Evaluation of user-prosthesis-interfaces for sEMG-based multifunctional prosthetic hands. Sensors (Basel).

[3] Hamet P, Tremblay J (2017). Artificial intelligence in medicine. Metabolism.

[4] Choi RY, Coyner AS, Kalpathy-Cramer J, Chiang MF, Campbell JP (2020). Introduction to machine learning, neural networks, and deep learning. Transl Vis Sci Technol.

[5] LeCun Y, Bengio Y, Hinton G (2015). Deep learning. Nature.

[6] Noorbakhsh-Sabet N, Zand R, Zhang Y, Abedi V (2019). Artificial intelligence transforms the future of health care. Am J Med.

[7] De Moor G, Sundgren M, Kalra D, Schmidt A, Dugas M, Claerhout B, Karakoyun T, Ohmann C, Lastic PY, Ammour N, Kush R, Dupont D, Cuggia M, Daniel C, Thienpont G, Coorevits P (2015). Using electronic health records for clinical research: the case of the EHR4CR project. J Biomed Inform.

[8] Nelson GS (2019). Bias in artificial intelligence. N C Med J.

[9] Yoon DY, Mansukhani NA, Stubbs VC, Helenowski IB, Woodruff TK, Kibbe MR (2014). Sex bias exists in basic science and translational surgical research. Surgery.

[10] Klare BF, Burge MJ, Klontz JC, Vorder Bruegge RW, Jain AK (2012). Face recognition performance: role of demographic information. IEEE Trans Inform Forensic Secur.

[11] Caliskan A, Bryson JJ, Narayanan A (2017). Semantics derived automatically from language corpora contain human-like biases. Science.

[12] (2018). Towards trustable machine learning. Nat Biomed Eng.

[13] Tomašev N, Glorot X, Rae JW, Zielinski M, Askham H, Saraiva A, Mottram A, Meyer C, Ravuri S, Protsyuk I, Connell A, Hughes CO, Karthikesalingam A, Cornebise J, Montgomery H, Rees G, Laing C, Baker CR, Peterson K, Reeves R, Hassabis D, King D, Suleyman M, Back T, Nielson C, Ledsam JR, Mohamed S (2019). A clinically applicable approach to continuous prediction of future acute kidney injury. Nature.

[14] Zhu C, Boutros PC (2021). Sex differences in cancer genomes: much learned, more unknown. Endocrinology.

[15] The Lancet Neurology (2019). A spotlight on sex differences in neurological disorders. Lancet Neurol.

[16] Takahashi T, Ellingson MK, Wong P, Israelow B, Lucas C, Klein J, Silva J, Mao T, Oh JE, Tokuyama M, Lu P, Venkataraman A, Park A, Liu F, Meir A, Sun J, Wang EY, Casanovas-Massana A, Wyllie AL, Vogels CBF, Earnest R, Lapidus S, Ott IM, Moore AJ, Shaw A, Fournier JB, Odio CD, Farhadian S, Dela Cruz C, Grubaugh ND, Schulz WL, Ring AM, Ko AI, Omer SB, Iwasaki A, Yale IMPACT Research Team (2020). Sex differences in immune responses that underlie COVID-19 disease outcomes. Nature.

[17] Mauvais-Jarvis F (2018). Gender differences in glucose homeostasis and diabetes. Physiol Behav.

[18] Shang D, Wang L, Klionsky DJ, Cheng H, Zhou R (2021). Sex differences in autophagy-mediated diseases: toward precision medicine. Autophagy.

[19] Brady E, Nielsen MW, Andersen JP, Oertelt-Prigione S (2021). Lack of consideration of sex and gender in COVID-19 clinical studies. Nat Commun.

[20] Gilpin LH, Bau D, Yuan BZ, Bajwa A, Specter M, Kagal L Explaining explanations: an overview of interpretability of machine learning. arXiv.

[21] Yang G, Ye Q, Xia J (2022). Unbox the black-box for the medical explainable AI via multi-modal and multi-centre data fusion: a mini-review, two showcases and beyond. Inf Fusion.

[22] Montavon G, Binder A, Lapuschkin S, Samek W, Müller KR, Samek W, Montavon G, Vedaldi A, Hansen LK, Müller KR (2019). Layer-wise relevance propagation: an overview. Explainable AI: Interpreting, Explaining and Visualizing Deep Learning.

[23] Ehsan U, Harrison L, Chan L, Riedl MO Rationalization: a neural machine translation approach to generating natural language explanations. arXiv.

[24] Friedrich S, Antes G, Behr S, Binder H, Brannath W, Dumpert F, Ickstadt K, Kestler HA, Lederer J, Leitgöb H, Pauly M, Steland A, Wilhelm A, Friede T (2021). Is there a role for statistics in artificial intelligence?. Adv Data Anal Classif.

[25] Harris PA, Taylor R, Minor BL, Elliott V, Fernandez M, O'Neal L, McLeod L, Delacqua G, Delacqua F, Kirby J, Duda SN, REDCap Consortium (2019). The REDCap consortium: building an international community of software platform partners. J Biomed Inform.

[26] Harris PA, Taylor R, Thielke R, Payne J, Gonzalez N, Conde JG (2009). Research electronic data capture (REDCap): a metadata-driven methodology and workflow process for providing translational research informatics support. J Biomed Inform.

[27] Barredo Arrieta A, Díaz-Rodríguez N, Del Ser J, Bennetot A, Tabik S, Barbado A, Garcia S, Gil-Lopez S, Molina D, Benjamins R, Chatila R, Herrera F (2020). Explainable artificial intelligence (XAI): concepts, taxonomies, opportunities and challenges toward responsible AI. Inf Fusion.

[28] Mons B, Neylon C, Velterop J, Dumontier M, da Silva Santos LOB, Wilkinson MD (2017). Cloudy, increasingly FAIR; revisiting the FAIR data guiding principles for the European open science cloud. Inf Serv Use.

[29] Manrai AK, Patel CJ, Ioannidis JPA (2018). In the era of precision medicine and big data, who is normal?. JAMA.

[30] Chen I, Johansson FD, Sontag D Why is my classifier discriminatory?. arXiv.

[31] Wilkinson MD, Dumontier M, Aalbersberg IJJ, Appleton G, Axton M, Baak A, Blomberg N, Boiten JW, da Silva Santos LB, Bourne PE, Bouwman J, Brookes AJ, Clark T, Crosas M, Dillo I, Dumon O, Edmunds S, Evelo CT, Finkers R, Gonzalez-Beltran A, Gray AJG, Groth P, Goble C, Grethe JS, Heringa J, 't Hoen PAC, Hooft R, Kuhn T, Kok R, Kok J, Lusher SJ, Martone ME, Mons A, Packer AL, Persson B, Rocca-Serra P, Roos M, van Schaik R, Sansone SA, Schultes E, Sengstag T, Slater T, Strawn G, Swertz MA, Thompson M, van der Lei J, van Mulligen E, Velterop J, Waagmeester A, Wittenburg P, Wolstencroft K, Zhao J, Mons B (2016). The FAIR guiding principles for scientific data management and stewardship. Sci Data.

[32] (1991). IEEE Standard Computer Dictionary: A Compilation of IEEE Standard Computer Glossaries. IEEE Std 610.

[33] Benson T, Grieve G (2016). Principles of Health Interoperability: SNOMED CT, HL7 and FHIR, 3rd Edition.

[34] Arvanitis TN (2014). Semantic interoperability in healthcare. Stud Health Technol Inform.

[35] Gansel X, Mary M, van Belkum A (2019). Semantic data interoperability, digital medicine, and e-health in infectious disease management: a review. Eur J Clin Microbiol Infect Dis.

[36] Franconi F, Campesi I (2014). Pharmacogenomics, pharmacokinetics and pharmacodynamics: interaction with biological differences between men and women. Br J Pharmacol.

[37] Reddy S, Allan S, Coghlan S, Cooper P (2020). A governance model for the application of AI in health care. J Am Med Inform Assoc.

[38] Wang HE, Landers M, Adams R, Subbaswamy A, Kharrazi H, Gaskin DJ, Saria S (2022). A bias evaluation checklist for predictive models and its pilot application for 30-day hospital readmission models. J Am Med Inform Assoc.

[39] McCradden MD, Joshi S, Anderson JA, Mazwi M, Goldenberg A, Zlotnik Shaul R (2020). Patient safety and quality improvement: ethical principles for a regulatory approach to bias in healthcare machine learning. J Am Med Inform Assoc.

[40] Wiegand T, Lee N, Pujari S, Singh M, Xu S, Kuglitsch M, Lecoultre M, Riviere-Cinnamond A, Weicken E, Wenzel M, Leite AW, Campos S, Quast B (2019). Whitepaper for the ITU/WHO focus group on artificial intelligence for health. International Telecommunication Union.

[41] (2019). Global gender gap report 2020. World Economic Forum.

[42] Serdar CC, Cihan M, Yücel D, Serdar MA (2021). Sample size, power and effect size revisited: simplified and practical approaches in pre-clinical, clinical and laboratory studies. Biochem Med (Zagreb).

[43] Challen R, Denny J, Pitt M, Gompels L, Edwards T, Tsaneva-Atanasova K (2019). Artificial intelligence, bias and clinical safety. BMJ Qual Saf.

[44] Tavakol M, Dennick R (2011). Making sense of Cronbach's alpha. Int J Med Educ.

[45] (2022). Analysis on survey bias in AI. GitHub.

