# Supplementary material for: Artificial Intelligence Bias in Health Care: Web-Based Survey
Source: J Med Internet Res. 2023 Jun 22;25:e41089. doi: 10.2196/41089 (PMC10337406; doi:10.2196/41089)
Supplement: Multimedia Appendix 1 [file jmir_v25i1e41089_app1.pdf]

Codebook

Data Dictionary Codebook

20.09.2021 12:12

Collapse all instruments

#	Variable / Field Name	Field Label<br>*Field Note*	Field Attributes (Field Type, Validation, Choices, Calculations, etc.)
Instrument: **Sprachauswahl** (sprachauswahl) Enabled as survey			Collapse
1	id	ID	text<br>Field Annotation: @HIDDEN
2	language	Bitte wählen Sie Ihre Sprache/ Please choose your language:	radio, Required<br>1 Deutsch<br>2 English
3	sprachauswahl_complete	Section Header: *Form Status*<br>Complete?	dropdown<br>0 Incomplete<br>1 Unverified<br>2 Complete
Instrument: **Fragebogen Fairness Und Ki** (fragebogen_fairness_und_ki) Enabled as survey			Collapse
4	quelle	Wie sind Sie auf diesen Fragebogen aufmerksam geworden?	radio<br>1 Twitter<br>2 Linkedin<br>3 Emailverteiler der eigenen Einrichtung<br>4 Persönliches Anschreiben<br>5 Andere (bitte angeben)<br>6 Keine Angabe<br><br>Custom alignment: LV
5	quelle_andere<br><br>Show the field ONLY if:<br>[quelle] = '5'	Wie sind Sie auf diesen Fragebogen aufmerksam geworden:	text

6	arbeitsland	In welchem Land arbeiten Sie?	dropdown<br>1 Belgien<br>2 Deutschland<br>3 Frankreich<br>4 Italien<br>5 Luxembourg<br>6 Niederlande<br>7 Dänemark<br>8 Irland<br>9 Griechenland<br>10 Portugal<br>11 Spanien<br>12 Finnland<br>13 Österreich<br>14 Schweden<br>15 Estland<br>16 Lettland<br>17 Litauen<br>18 Malta<br>19 Polen<br>20 Slowakei<br>21 Slowenien<br>22 Tschechien<br>23 Ungarn<br>24 Zypern<br>25 Bulgarien<br>26 Rumänien<br>27 Kroatien<br>28 Anderes Land (bitte angeben)<br>29 Keine Angabe
7	arbeitsland_andere<br>Show the field ONLY if:<br>[arbeitsland] = '28'	In welchem anderen Land arbeiten Sie:	text
8	arbeitgeber	In welcher Firma/Institution arbeiten Sie?	text
9	geschlecht	Bitte geben Sie ihr Geschlecht an:	radio<br>1 Weiblich<br>2 Männlich<br>3 Divers<br>4 Unbestimmt<br>5 Keine Angabe<br>Custom alignment: LH
10	alter	Wie alt sind Sie?<br>*Jahre*	text (number, Min: 15, Max: 100)
11	sektor	In welchem Sektor sind Sie tätig?	radio<br>1 Wissenschaft<br>2 Klinische Tätigkeit<br>3 Wirtschaft<br>4 Andere (bitte angeben)<br>5 Keine Angabe<br>Custom alignment: LV
12	sektor_andere<br>Show the field ONLY if:<br>[sektor] = '4'	In welchem anderen Sektor sind Sie tätig:	text

13	ki_entwicklung	Sind Sie derzeit an einer KI-Entwicklung beteiligt?	radio, Required<br>1 Ja<br>2 Nein, aber in der Vergangenheit<br>3 Nein, aber ist in Planung<br>4 Nein, noch nie<br>5 Keine Angabe<br><br>Custom alignment: LV
14	kommentar<br><br>Show the field ONLY if:<br>[ki_entwicklung] = '4' or [ki_entwicklung] = '5'	Haben Sie generelle Anmerkungen zum Fragebogen?	notes
15	fragebogen_fairness_und_ki_complete	Section Header: *Form Status*<br>Complete?	dropdown<br>0 Incomplete<br>1 Unverified<br>2 Complete
Instrument: **Fragebogen Fairness Und Ki 2** (fragebogen_fairness_und_ki_2) Enabled as survey			Collapse
16	ki_entwicklungsphase	In welcher Phase entwickeln oder haben Sie KI-Projekte entwickelt?	checkbox<br>1 ki_entwicklungsphase___1 Projektplanung<br>2 ki_entwicklungsphase___2 Datenerfassung/-aufbereitung<br>3 ki_entwicklungsphase___3 Datenannotation<br>4 ki_entwicklungsphase___4 Identifizierung der KI-Algorithmen<br>5 ki_entwicklungsphase___5 Training und Optimierung<br>6 ki_entwicklungsphase___6 Ertesten der KI-Algorithmen in der Praxis<br>7 ki_entwicklungsphase___7 Andere (bitte angeben)<br>8 ki_entwicklungsphase___8 Keine Angabe
17	ki_entwicklungsphase_ander<br><br>Show the field ONLY if:<br>[ki_entwicklungsphase(7)] = '1'	In welcher anderen Phase der Entwicklung befinden Sie sich in einem derzeitigen Projekt:	text

<table>
<tr><td>18</td><td>med_fachbereich</td><td>In welchem medizinischen Fachbereich entwickeln Sie KI-Anwendungen?</td><td>checkbox
<table>
<tr><td>1</td><td>med_fachbereich___1</td><td>Allgemeinmedizin</td></tr>
<tr><td>2</td><td>med_fachbereich___2</td><td>Anästhesiologie</td></tr>
<tr><td>3</td><td>med_fachbereich___3</td><td>Anatomie</td></tr>
<tr><td>4</td><td>med_fachbereich___4</td><td>Arbeitsmedizin</td></tr>
<tr><td>5</td><td>med_fachbereich___5</td><td>Augenheilkunde</td></tr>
<tr><td>6</td><td>med_fachbereich___6</td><td>Biochemie</td></tr>
<tr><td>7</td><td>med_fachbereich___7</td><td>Chirurgie</td></tr>
<tr><td>8</td><td>med_fachbereich___8</td><td>Frauenheilkunde und Geburtshilfe</td></tr>
<tr><td>9</td><td>med_fachbereich___9</td><td>Hals-Nasen-Ohrenheilkunde</td></tr>
<tr><td>10</td><td>med_fachbereich___10</td><td>Haut- und Geschlechtskrankheiten</td></tr>
<tr><td>11</td><td>med_fachbereich___11</td><td>Humangenetik</td></tr>
<tr><td>12</td><td>med_fachbereich___12</td><td>Hygiene und Umweltmedizin</td></tr>
<tr><td>13</td><td>med_fachbereich___13</td><td>Innere Medizin</td></tr>
<tr><td>14</td><td>med_fachbereich___14</td><td>Kinder- und Jugendmedizin</td></tr>
<tr><td>15</td><td>med_fachbereich___15</td><td>Kinder- und Jugendpsychiatrie und -psychotherapie</td></tr>
<tr><td>16</td><td>med_fachbereich___16</td><td>Laboratoriumsmedizin</td></tr>
<tr><td>17</td><td>med_fachbereich___17</td><td>Mikrobiologie, Virologie und Infektionsepidemiologie</td></tr>
<tr><td>18</td><td>med_fachbereich___18</td><td>Mund-Kiefer-Gesichtschirurgie</td></tr>
<tr><td>19</td><td>med_fachbereich___19</td><td>Neurochirurgie</td></tr>
<tr><td>20</td><td>med_fachbereich___20</td><td>Neurologie</td></tr>
<tr><td>21</td><td>med_fachbereich___21</td><td>Nuklearmedizin</td></tr>
<tr><td>22</td><td>med_fachbereich___22</td><td>Öffentliches Gesundheitswesen</td></tr>
<tr><td>23</td><td>med_fachbereich___23</td><td>Pathologie</td></tr>
<tr><td>24</td><td>med_fachbereich___24</td><td>Pharmakologie</td></tr>
<tr><td>25</td><td>med_fachbereich___25</td><td>Phoniatrie und Pädaudiologie</td></tr>
<tr><td>26</td><td>med_fachbereich___26</td><td>Physikalische und Rehabilitative Medizin</td></tr>
<tr><td>27</td><td>med_fachbereich___27</td><td>Physiologie</td></tr>
<tr><td>28</td><td>med_fachbereich___28</td><td>Psychiatrie und Psychotherapie</td></tr>
<tr><td>29</td><td>med_fachbereich___29</td><td>Psychosomatische Medizin und Psychotherapie</td></tr>
<tr><td>30</td><td>med_fachbereich___30</td><td>Radiologie</td></tr>
<tr><td>31</td><td>med_fachbereich___31</td><td>Rechtsmedizin</td></tr>
<tr><td>32</td><td>med_fachbereich___32</td><td>Strahlentherapie</td></tr>
<tr><td>33</td><td>med_fachbereich___33</td><td>Transfusionsmedizin</td></tr>
<tr><td>34</td><td>med_fachbereich___34</td><td>Urologie</td></tr>
<tr><td>35</td><td>med_fachbereich___35</td><td>Medizininformatik/ Digitale Medizin</td></tr>
<tr><td>36</td><td>med_fachbereich___36</td><td>Andere (bitte angeben)</td></tr>
<tr><td>37</td><td>med_fachbereich___37</td><td>Keine Angabe</td></tr>
</table></td></tr>
<tr><td>19</td><td>med_fachbereich_andere
Show the field ONLY if:
[med_fachbereich(36)] = '1'</td><td>In welchem anderen medizinischen Fachbereich entwickeln Sie KI-Anwendungen:</td><td>text</td></tr>
</table>

<table>
<tr><td>20</td><td>ki_art</td><td>In welchem der folgenden KI Bereiche entwickeln Sie?</td><td>checkbox<table><tr><td>1</td><td>ki_art___1</td><td>Maschinelles Lernen</td></tr><tr><td>2</td><td>ki_art___2</td><td>Deep Learning</td></tr><tr><td>3</td><td>ki_art___3</td><td>Andere Form von KI</td></tr><tr><td>4</td><td>ki_art___4</td><td>Keine Angabe</td></tr></table></td></tr>
<tr><td>21</td><td>ki_art_ml
Show the field ONLY if:
[ki_art(1)] = '1'</td><td>Bitte spezifizieren Sie die Art von Maschinellem Lernen (ML) welche Sie entwickeln:</td><td>checkbox<table><tr><td>1</td><td>ki_art_ml___1</td><td>Supervised ML</td></tr><tr><td>2</td><td>ki_art_ml___2</td><td>Semi-supervised ML</td></tr><tr><td>3</td><td>ki_art_ml___3</td><td>Unsupervised ML</td></tr><tr><td>4</td><td>ki_art_ml___4</td><td>Reinforcement learning</td></tr><tr><td>5</td><td>ki_art_ml___5</td><td>Andere</td></tr><tr><td>6</td><td>ki_art_ml___6</td><td>Keine Angabe</td></tr></table></td></tr>
<tr><td>22</td><td>ki_art_dl
Show the field ONLY if:
[ki_art(2)] = '1'</td><td>Bitte spezifizieren Sie die Art von Deep Learning welche Sie entwickeln:</td><td>checkbox<table><tr><td>1</td><td>ki_art_dl___1</td><td>Convolutional Networks</td></tr><tr><td>2</td><td>ki_art_dl___2</td><td>Recurrent neural Networks</td></tr><tr><td>3</td><td>ki_art_dl___3</td><td>Autoencoders</td></tr><tr><td>4</td><td>ki_art_dl___4</td><td>Andere</td></tr><tr><td>5</td><td>ki_art_dl___5</td><td>Keine Angabe</td></tr></table></td></tr>
<tr><td>23</td><td>ki_bereich</td><td>In welchem der folgenden KI Bereiche entwickeln Sie?</td><td>checkbox<table><tr><td>1</td><td>ki_bereich___1</td><td>Natural Language Processing</td></tr><tr><td>2</td><td>ki_bereich___2</td><td>Clinical Decision Support</td></tr><tr><td>3</td><td>ki_bereich___3</td><td>Bildverarbeitung</td></tr><tr><td>4</td><td>ki_bereich___4</td><td>Computer Vision (Bsp. Objekterkennung)</td></tr><tr><td>5</td><td>ki_bereich___5</td><td>Robotics</td></tr><tr><td>6</td><td>ki_bereich___6</td><td>Andere (bitte angeben)</td></tr><tr><td>7</td><td>ki_bereich___7</td><td>Keine Angabe</td></tr></table></td></tr>
<tr><td>24</td><td>ki_bereich_andere
Show the field ONLY if:
[ki_bereich(6)] = '1'</td><td>In welchem anderen KI Bereich entwickeln Sie:</td><td>text</td></tr>
<tr><td>25</td><td>bias_ki</td><td>Wie vertraut sind sie mit Bias in der KI?</td><td>radio<table><tr><td>1</td><td>Ich habe von Bias in der KI noch nichts gehört</td></tr><tr><td>2</td><td>Ich habe von Bias gehört, aber könnte keine konkreten Beispiele nennen</td></tr><tr><td>3</td><td>Ich habe von Bias in der KI gehört und kenne spezifische Use-cases</td></tr></table></td></tr>
<tr><td>26</td><td>bias_feld</td><td>Wo kann Ihrer Meinung nach Bias in KI auftreten?</td><td>checkbox<table><tr><td>1</td><td>bias_feld___1</td><td>Methodik der Algorithmen</td></tr><tr><td>2</td><td>bias_feld___2</td><td>Gesellschaftliche Faktoren</td></tr><tr><td>3</td><td>bias_feld___3</td><td>Bias basierend auf Datenvalidierungs- oder Datensicherheitsmethodik</td></tr><tr><td>4</td><td>bias_feld___4</td><td>Trifft nicht zu</td></tr><tr><td>5</td><td>bias_feld___5</td><td>Andere (bitte angeben)</td></tr><tr><td>6</td><td>bias_feld___6</td><td>Keine der genannten</td></tr></table></td></tr>
<tr><td>27</td><td>bias_feld_andere
Show the field ONLY if:
[bias_feld(5)] = '1'</td><td>Wo kann Ihrer Meinung nach Bias in KI auftreten? Anderen Bereich bitte angeben:</td><td>text</td></tr>
<tr><td>28</td><td>ki_trainingsdaten</td><td>Welche Daten nutzen Sie, um KI-Algorithmen zu trainieren?</td><td>checkbox<table><tr><td>1</td><td>ki_trainingsdaten___1</td><td>Bilddaten</td></tr><tr><td>2</td><td>ki_trainingsdaten___2</td><td>Audiodaten</td></tr><tr><td>3</td><td>ki_trainingsdaten___3</td><td>Textdaten</td></tr><tr><td>4</td><td>ki_trainingsdaten___4</td><td>Keine Angabe</td></tr></table></td></tr>
</table>

29	ki_daten_quelle	Woher stammen die von Ihnen verwendeten Daten?	checkbox<br>1 ki_daten_quelle___1 Wearables<br>2 ki_daten_quelle___2 Register<br>3 ki_daten_quelle___3 Datenbank aus einem Zentrum<br>4 ki_daten_quelle___4 Multizentrische Datenbank<br>5 ki_daten_quelle___5 Andere (bitte angeben)<br>6 ki_daten_quelle___6 Keine Angabe
30	ki_daten_quelle_andere<br>Show the field ONLY if:<br>[ki_daten_quelle(5)] = '1'	Die verwendeten Daten stammen aus:	text
31	nat_internat_daten	Arbeiten Sie mit nationalen oder internationalen Daten?	radio<br>1 Nationale Daten<br>2 Internationale Daten<br>3 Nationale und internationale Daten<br>4 Keine Angabe
32	standard_daten	Denken Sie, die Verwendung standardisierter Daten (internationale semantische und syntaktische Standards wie HL7 FHIR, SNOMED CT) zum Trainieren der Algorithmen kann Bias verhindern?	radio<br>1 Ja<br>2 Nein<br>3 Keine Angabe
33	massnahmen_bias	Mit welchen Maßnahmen zur Vermeidung von Bias in der KI sind Sie vertraut?	checkbox<br>1 massnahmen_bias___1 Verwendung von Explainable Artificial Intelligence (XAI)/ Erklärbare KI<br>2 massnahmen_bias___2 Erhebung von soziodemographischen Datenpunkten<br>3 massnahmen_bias___3 Statistische Analyse<br>4 massnahmen_bias___4 Programme zur Bewertung der Fairness von KI<br>5 massnahmen_bias___5 Mir sind keine bekannt<br>6 massnahmen_bias___6 Andere (bitte angeben)
34	massnahmen_bias_andere<br>Show the field ONLY if:<br>[massnahmen_bias(6)] = '1'	Mit nennen sie die anderen Maßnahmen zur Vermeidung von Bias in der KI, mit denen Sie vertraut sind:	text
35	soziodem_daten	Welche der folgenden soziodemographischen Datenpunkte würden Sie zur Vermeidung von Bias in der KI erheben?	checkbox<br>1 soziodem_daten___1 Biologisches Geschlecht<br>2 soziodem_daten___2 Soziales Geschlecht<br>3 soziodem_daten___3 Alter<br>4 soziodem_daten___4 Herkunft<br>5 soziodem_daten___5 Andere (bitte angeben)<br>6 soziodem_daten___6 Keine<br>7 soziodem_daten___7 Keine Angabe
36	soziodem_daten_andere<br>Show the field ONLY if:<br>[soziodem_daten(5)] = '1'	Welche anderen soziodemographischen Datenpunkte würden Sie zur Vermeidung von Bias in der KI erheben:	text
37	soziodem_verwendung<br>Show the field ONLY if:<br>[soziodem_daten(1)] = '1' or [soziodem_daten(2)] = '1' or [soziodem_daten(3)] = '1' or [soziodem_daten(4)] = '1' or [soziodem_daten(5)] = '1'	Wofür würden Sie die von Ihnen genannten soziodemographischen Datenpunkte verwenden?	checkbox<br>1 soziodem_verwendung___1 Modellierung<br>2 soziodem_verwendung___2 Analyse<br>3 soziodem_verwendung___3 Datenerhebung<br>4 soziodem_verwendung___4 Keine Angabe

38	fairness_ki	Wie schätzen Sie die Fairness von KI in Ihrem Bereich der KI-Entwicklung ein?<br>(Werden bestimmte Personengruppen nicht gleich berücksichtigt/behandelt?)	radio<br>1 Gar nicht fair<br>2 Kaum fair<br>3 Mäßig fair<br>4 Fair<br>5 Sehr fair<br>6 Keine Angabe
39	fairness_ki_block<br><br>Show the field ONLY if:<br>[fairness_ki] = '1' or [fairness_ki] = '2' or [fairness_ki] = '3' or [fairness_ki] = '4' or [fairness_ki] = '6'	Was verhindert Ihrer Meinung nach faire KI in Ihrem Anwendungsbereich?	checkbox<br>1 fairness_ki_block___1 Mangel an Ressourcen<br>2 fairness_ki_block___2 Mangelnde Unterstützung von Vorgesetzten/ der Einrichtung<br>3 fairness_ki_block___3 Mangelndes Wissen/ Know-how<br>4 fairness_ki_block___4 Mangel an fairen Daten<br>5 fairness_ki_block___5 Mangel an Guidelines/Vorgaben für faire KI<br>6 fairness_ki_block___6 Andere (bitte angeben)<br>7 fairness_ki_block___7 Keine Angabe
40	fairness_ki_block_andere<br><br>Show the field ONLY if:<br>[fairness_ki_block(6)] = '1'	Welche anderen Faktoren verhindern Ihrer Meinung nach faire KI in Ihrem Anwendungsbereich:	text
41	kommentar_1	Haben Sie generelle Anmerkungen zum Fragebogen?	notes
42	fragebogen_fairness_und_ki_2_complete	Section Header: *Form Status*<br>Complete?	dropdown<br>0 Incomplete<br>1 Unverified<br>2 Complete
Instrument: **Questionnaire Fairness And Ai** (questionnaire_fairness_and_ai) Enabled as survey Collapse			
43	quelle_1	How did this questionnaire reach you?	radio<br>1 Twitter<br>2 Linkedin<br>3 Email distribution list of my institution<br>4 Personal contact<br>5 Other (please specify)<br>6 Not specified<br><br>Custom alignment: LV
44	quelle_andere_1<br><br>Show the field ONLY if:<br>[quelle_1] = '5'	How did this questionnaire reach you:	text

45	arbeitsland_1	Which country do you work in?	dropdown<br>1 Belgium<br>2 Germany<br>3 France<br>4 Italy<br>5 Luxembourg<br>6 Netherlands<br>7 Denmark<br>8 Ireland<br>9 Greece<br>10 Portugal<br>11 Spain<br>12 Finland<br>13 Austria<br>14 Sweden<br>15 Estonia<br>16 Latvia<br>17 Lithuania<br>18 Malta<br>19 Poland<br>20 Slovakia<br>21 Slovenia<br>22 Czech Republic<br>23 Hungary<br>24 Cyprus<br>25 Bulgaria<br>26 Romania<br>27 Croatia<br>28 Other (please specify)<br>29 Not specified
46	arbeitsland_andere_1<br>Show the field ONLY if:<br>[arbeitsland_1] = "28"	Which country do you work in:	text
47	arbeitgeber_1	What is the name of your company/institution?	text
48	geschlecht_1	What is your gender?	radio<br>1 Female<br>2 Male<br>3 Diverse<br>4 Undefined<br>5 Not specified<br><br>Custom alignment: LH
49	alter_1	What is your age?<br>*years*	text (number, Min: 15, Max: 100)
50	sektor_1	In which area do you work in?	radio<br>1 Science<br>2 Clinical Work<br>3 Industry<br>4 Other (please specify)<br>5 Not specified<br><br>Custom alignment: LV
51	sektor_andere_1<br>Show the field ONLY if:<br>[sektor_1] = "4"	What is your work environment:	text

52	ki_entwicklung_1	Are you currently involved in AI-developments?	radio, Required<br>1 Yes<br>2 No, but I have worked on AI-projects in the past<br>3 No, but I am planning on it<br>4 No, never<br>5 Not specified<br><br>Custom alignment: LV
53	kommentar_2<br><br>Show the field ONLY if:<br>[ki_entwicklung_1] = '4' or [ki_entwicklung_1] = '5'	Do you have any comments you want to provide about the questionnaire?	text
54	questionnaire_fairness_and_ai_complete	Section Header: *Form Status*<br><br>Complete?	dropdown<br>0 Incomplete<br>1 Unverified<br>2 Complete

Instrument: **Questionnaire Fairness And Ai 2** (questionnaire_fairness_and_ai_2) Enabled as survey Collapse

55	ki_entwicklungsphase_1	At what stage are you or have you been developing AI-projects?	checkbox<br>1 ki_entwicklungsphase_1___1 Project planning<br>2 ki_entwicklungsphase_1___2 Data acquisition/ data preprocessing<br>3 ki_entwicklungsphase_1___3 Data annotation<br>4 ki_entwicklungsphase_1___4 Identification of AI algorithms<br>5 ki_entwicklungsphase_1___5 Training and optimization<br>6 ki_entwicklungsphase_1___6 Practical testing of AI algorithms<br>7 ki_entwicklungsphase_1___7 Other (please specify)<br>8 ki_entwicklungsphase_1___8 Not specified
56	ki_entwicklungsphase_ander_1<br><br>Show the field ONLY if:<br>[ki_entwicklungsphase_1(7)] = '1'	At what other stage are/were you developing:	text

<table>
<tr><td>57</td><td>med_fachbereich_1</td><td>In which medical specialty are you developing AI solutions?</td><td>checkbox
<table>
<tr><td>1</td><td>med_fachbereich_1___1</td><td>General Medicine/Family Medicine</td></tr>
<tr><td>2</td><td>med_fachbereich_1___2</td><td>Anesthesiology</td></tr>
<tr><td>3</td><td>med_fachbereich_1___3</td><td>Anatomy</td></tr>
<tr><td>4</td><td>med_fachbereich_1___4</td><td>Occupational medicine</td></tr>
<tr><td>5</td><td>med_fachbereich_1___5</td><td>Ophthalmology</td></tr>
<tr><td>6</td><td>med_fachbereich_1___6</td><td>Biochemistry</td></tr>
<tr><td>7</td><td>med_fachbereich_1___7</td><td>Surgery</td></tr>
<tr><td>8</td><td>med_fachbereich_1___8</td><td>Obstetrics and gynecology</td></tr>
<tr><td>9</td><td>med_fachbereich_1___9</td><td>Otorhinolaryngology</td></tr>
<tr><td>10</td><td>med_fachbereich_1___10</td><td>Dermatology</td></tr>
<tr><td>11</td><td>med_fachbereich_1___11</td><td>Genetics</td></tr>
<tr><td>12</td><td>med_fachbereich_1___12</td><td>Environmental health</td></tr>
<tr><td>13</td><td>med_fachbereich_1___13</td><td>Internal medicine</td></tr>
<tr><td>14</td><td>med_fachbereich_1___14</td><td>Pediatrics</td></tr>
<tr><td>15</td><td>med_fachbereich_1___15</td><td>Child and adolescent psychiatry</td></tr>
<tr><td>16</td><td>med_fachbereich_1___16</td><td>Laboratory medicine</td></tr>
<tr><td>17</td><td>med_fachbereich_1___17</td><td>Clinical microbiology/ Infectious disease</td></tr>
<tr><td>18</td><td>med_fachbereich_1___18</td><td>Oral and maxillofacial surgery</td></tr>
<tr><td>19</td><td>med_fachbereich_1___19</td><td>Neurosurgery</td></tr>
<tr><td>20</td><td>med_fachbereich_1___20</td><td>Neurology</td></tr>
<tr><td>21</td><td>med_fachbereich_1___21</td><td>Nuclear medicine</td></tr>
<tr><td>22</td><td>med_fachbereich_1___22</td><td>Public Health</td></tr>
<tr><td>23</td><td>med_fachbereich_1___23</td><td>Pathology</td></tr>
<tr><td>24</td><td>med_fachbereich_1___24</td><td>Pharmacology</td></tr>
<tr><td>25</td><td>med_fachbereich_1___25</td><td>Phoniatrics and paediatric audiology</td></tr>
<tr><td>26</td><td>med_fachbereich_1___26</td><td>Physical medicine and rehabilitation</td></tr>
<tr><td>27</td><td>med_fachbereich_1___27</td><td>Physiology</td></tr>
<tr><td>28</td><td>med_fachbereich_1___28</td><td>Psychiatry/ Psychotherapy</td></tr>
<tr><td>29</td><td>med_fachbereich_1___29</td><td>Psychosomatics</td></tr>
<tr><td>30</td><td>med_fachbereich_1___30</td><td>Radiology</td></tr>
<tr><td>31</td><td>med_fachbereich_1___31</td><td>Forensic medicine</td></tr>
<tr><td>32</td><td>med_fachbereich_1___32</td><td>Radiotherapy</td></tr>
<tr><td>33</td><td>med_fachbereich_1___33</td><td>Transfusion medicine</td></tr>
<tr><td>34</td><td>med_fachbereich_1___34</td><td>Urology</td></tr>
<tr><td>35</td><td>med_fachbereich_1___35</td><td>Medical Informatics/ Digital Medicine</td></tr>
<tr><td>36</td><td>med_fachbereich_1___36</td><td>Other (please specify)</td></tr>
<tr><td>37</td><td>med_fachbereich_1___37</td><td>Not specified</td></tr>
</table></td></tr>
<tr><td>58</td><td>med_fachbereich_andere_1
Show the field ONLY if: [med_fachbereich_1(36)] = '1'</td><td>In which other medical specialty are you developing AI solutions:</td><td>text</td></tr>
<tr><td>59</td><td>ki_art_1</td><td>In which of the following areas of AI are you developing?</td><td>checkbox
<table>
<tr><td>1</td><td>ki_art_1___1</td><td>Machine Learning</td></tr>
<tr><td>2</td><td>ki_art_1___2</td><td>Deep Learning</td></tr>
<tr><td>3</td><td>ki_art_1___3</td><td>Other type of AI</td></tr>
<tr><td>4</td><td>ki_art_1___4</td><td>Not specified</td></tr>
</table></td></tr>
</table>

60	ki_art_ml_1<br>Show the field ONLY if:<br>[ki_art_1(1)] = '1'	Please specify the type of Machine Learning (ML) you are developing:	checkbox<br>1 ki_art_ml_1___1 Supervised ML<br>2 ki_art_ml_1___2 Semi-supervised ML<br>3 ki_art_ml_1___3 Unsupervised ML<br>4 ki_art_ml_1___4 Reinforcement learning<br>5 ki_art_ml_1___5 Other<br>6 ki_art_ml_1___6 Not specified
61	ki_art_dl_1<br>Show the field ONLY if:<br>[ki_art_1(2)] = '1'	Please specify the type of Deep Learning you are developing:	checkbox<br>1 ki_art_dl_1___1 Convolutional Networks<br>2 ki_art_dl_1___2 Recurrent neural Networks<br>3 ki_art_dl_1___3 Autoencoders<br>4 ki_art_dl_1___4 Other<br>5 ki_art_dl_1___5 Not specified
62	ki_bereich_1	In which of the following areas of AI are you developing?	checkbox<br>1 ki_bereich_1___1 Natural Language Processing<br>2 ki_bereich_1___2 Clinical Decision Support<br>3 ki_bereich_1___3 Image processing<br>4 ki_bereich_1___4 Computer vision (e.g. object recognition)<br>5 ki_bereich_1___5 Robotics<br>6 ki_bereich_1___6 Other (please specify)<br>7 ki_bereich_1___7 Not specified
63	ki_bereich_andere_1<br>Show the field ONLY if:<br>[ki_bereich_1(6)] = '1'	Which other area of AI do your development efforts fall into:	text
64	bias_ki_1	How familiar are you with biases in AI?	radio<br>1 I have never heard of biases in AI<br>2 I have heard of biases in AI but I can not think of concrete examples<br>3 I have heard of biases in AI and I do know specific use cases
65	bias_feld_1	Where do you think biases in AI can occur?	checkbox<br>1 bias_feld_1___1 Methodology of algorithms<br>2 bias_feld_1___2 Societal factors<br>3 bias_feld_1___3 Bias due to data validation or data security<br>4 bias_feld_1___4 Not specified<br>5 bias_feld_1___5 Other (please specify)<br>6 bias_feld_1___6 None of the above
66	bias_feld_andere_1<br>Show the field ONLY if:<br>[bias_feld_1(5)] = '1'	Where else do you think biases in AI can occur?	text
67	ki_trainingsdaten_1	What data do you use to train AI algorithms?	checkbox<br>1 ki_trainingsdaten_1___1 Image data<br>2 ki_trainingsdaten_1___2 Audio data<br>3 ki_trainingsdaten_1___3 Text data<br>4 ki_trainingsdaten_1___4 Not specified
68	ki_daten_quelle_1	What is the origin of your data?	checkbox<br>1 ki_daten_quelle_1___1 Wearables<br>2 ki_daten_quelle_1___2 Registries<br>3 ki_daten_quelle_1___3 Database from one center<br>4 ki_daten_quelle_1___4 Multi-center database<br>5 ki_daten_quelle_1___5 Other (please specify)<br>6 ki_daten_quelle_1___6 Not specified

69	ki_daten_quelle_andere_1<br><br>Show the field ONLY if:<br>[ki_daten_quelle_1(5)] = '1'	Please specify the origin of your data:	text
70	nat_internat_daten_1	Do you work with national or international data?	radio<br>1 National data<br>2 International data<br>3 National and international data<br>4 Not specified
71	standard_daten_1	Do you think that using standardized data (international semantic and syntactic standards such as HL7 FHIR, SNOMED CT) when training AI algorithms can prevent bias?	radio<br>1 Yes<br>2 No<br>3 Not specified
72	massnahmen_bias_1	Do you know any of the following preventive measures to avoid bias in AI applications?	checkbox<br>1 massnahmen_bias_1___1 Employ Explainable Artificial Intelligence (XAI)<br>2 massnahmen_bias_1___2 Collecting sociodemographic data points<br>3 massnahmen_bias_1___3 Statistical analysis<br>4 massnahmen_bias_1___4 Software evaluating fairness in AI<br>5 massnahmen_bias_1___5 I do not know any of them<br>6 massnahmen_bias_1___6 Other (please specify)
73	massnahmen_bias_andere_1<br><br>Show the field ONLY if:<br>[massnahmen_bias_1(6)] = '1'	Please specify the other preventive measure(s) you know to avoid bias in AI applications:	text
74	soziodem_daten_1	What sociodemographic data would you collect to prevent biases in AI?	checkbox<br>1 soziodem_daten_1___1 Biological gender<br>2 soziodem_daten_1___2 Social gender<br>3 soziodem_daten_1___3 Age<br>4 soziodem_daten_1___4 Origin<br>5 soziodem_daten_1___5 Other (please specify)<br>6 soziodem_daten_1___6 None<br>7 soziodem_daten_1___7 Not specified
75	soziodem_daten_andere_1<br><br>Show the field ONLY if:<br>[soziodem_daten_1(5)] = '1'	What other sociodemographic data would you collect to prevent biases in AI?	text
76	soziodem_verwendung_1<br><br>Show the field ONLY if:<br>[soziodem_daten_1(1)] = '1' or<br>[soziodem_daten_1(2)] = '1' or<br>[soziodem_daten_1(3)] = '1' or<br>[soziodem_daten_1(4)] = '1' or<br>[soziodem_daten_1(5)] = '1'	What would you use the collected sociodemographic data for?	checkbox<br>1 soziodem_verwendung_1___1 AI modelling<br>2 soziodem_verwendung_1___2 Analysis<br>3 soziodem_verwendung_1___3 Data acquisition<br>4 soziodem_verwendung_1___4 Not specified
77	fairness_ki_1	How would you rate the level of fairness of AI in your AI development? (Are specific groups of people treated /considered differently from others?)	radio<br>1 Not fair at all<br>2 Barely fair<br>3 Moderately fair<br>4 Fair<br>5 Very fair<br>6 Not specified

78	fairness_ki_block_1<br>Show the field ONLY if:<br>[fairness_ki_1] = '1' or [fairness_ki_1] = '2' or [fairness_ki_1] = '3' or [fairness_ki_1] = '4' or [fairness_ki_1] = '6'	What do you think is preventing fair AI in your use case?	checkbox<br>1 fairness_ki_block_1___1 Lack of resources<br>2 fairness_ki_block_1___2 Lack of support from superiors/institution<br>3 fairness_ki_block_1___3 Lack of knowledge<br>4 fairness_ki_block_1___4 Lack of fair data<br>5 fairness_ki_block_1___5 Lack of guidelines/recommendations for fair AI<br>6 fairness_ki_block_1___6 Other (please specify)<br>7 fairness_ki_block_1___7 Not specified
79	fairness_ki_block_andere_1<br>Show the field ONLY if:<br>[fairness_ki_block_1(6)] = '1'	What other factor(s) do you think is/are preventing fair AI in your use case?	text
80	kommentar_3	Do you have any comments you want to provide about the questionnaire?	notes
81	questionnaire_fairness_and_ai_2_complete	Section Header: *Form Status*<br>Complete?	dropdown<br>0 Incomplete<br>1 Unverified<br>2 Complete
