# Supplementary material for: Artificial Intelligence Bias in Health Care: Web-Based Survey
Source: J Med Internet Res. 2023 Jun 22;25:e41089. doi: 10.2196/41089 (PMC10337406; doi:10.2196/41089)
Supplement: Multimedia Appendix 2 [file jmir_v25i1e41089_app2.docx]

**Supplemental Materials**

**Table S1: Type and area of AI used in AI projects. The table provides on overview of the absolute and relative (in brackets) number of responses to the two variables (questions) concerning the type of AI used in the AI project respondents were involved in and a specification of the area of AI of these projects.**

| **Type of AI used in AI project** |  | **N (%)** |
| --- | --- | --- |
| **Machine Learning** |  | 105 (70) |
| Supervised ML |  | 80 (53) |
| Semi-supervised ML |  | 42 (28) |
| Unsupervised ML |  | 41 (27) |
| Reinforcement learning |  | 21 (14) |
| Other |  | 7 (15) |
| Not specified |  | 6 (4) |
| **Deep Learning** |  | 86 (57) |
| Convolutional Networks |  | 71 (47) |
| Recurrent neural Networks |  | 40 (26) |
| Autoencoders |  | 30 (20) |
| Other |  | 26 (17) |
| Not specified |  | 0 (0) |
| **Other Type of AI** |  | 41 (27) |
| **Not specified** |  | 9 (6) |
| **Area of AI used in AI project** |  | **N (%)** |
| Natural Language Processing |  | 39 (26) |
| Clinical Decision Support |  | 53 (35) |
| Image processing |  | 64 (43) |
| Computer vision |  | 50 (33) |
| Robotics |  | 16 (11) |
| Other |  | 21 (14) |
| Not specified |  | 14 (9) |

**Table S2. “How would you rate the level of fairness of AI in your AI development” by gender, age and work environment, N (%).**

|  | **Not fair at all** | **Barely fair** | **Moderately fair** | **Fair** | **Very Fair** | **N.sp.** | **P value** | |
| --- | --- | --- | --- | --- | --- | --- | --- | --- |
| **Gender** |  |  |  |  |  |  |  | |
| Female | 0 (0) | 7 (16) | 13 (29) | 12 (27) | 6 (13) | 7 (16) | **0.174** | |
| Male | 2 (2) | 8 (8) | 37 (37) | 35 (35) | 13 (13) | 6 (6) |  |  |
| Diverse | 0 (0) | 1 (100) | 0 (0) | 0 (0) | 0 (0) | 0 (0) |  |  |
| Undefined | 0 (0) | 1 (50) | 1 (50) | 0 (0) | 0 (0) | 0 (0) |  |  |
| **Age (y)** |  |  |  |  |  |  |  | |
| <30 | 0 (0) | 7 (10) | 29 (42) | 21 (30) | 9 (13) | 3 (4) | **0.086** | |
| 30-40 | 0 (0) | 5 (10) | 12 (24) | 19 (39) | 6 (12) | 7 (14) |  |  |
| 40-50 | 1 (4) | 5 (21) | 9 (38) | 4 (17) | 3 (12) | 2 (8) |  |  |
| >50 | 1 (11) | 1 (11) | 1 (11) | 3 (33) | 1 (11) | 2 (22) |  |  |
| **Environment** |  |  |  |  |  |  |  | |
| Science | 0 (0) | 10 (10) | 39 (39) | 32 (32) | 10 (10) | 8 (8) | **0.020** | |
| Clinical Work | 1 (8) | 2 (17) | 3 (25) | 5 (42) | 1 (8) | 0 (0) |  |  |
| Industry | 0 (0) | 1 (5) | 5 (23) | 6 (27) | 6 (27) | 4 (18) |  |  |

n.sp.: not specified.
